# Corticosteroids for COVID-19-induced olfactory dysfunction: A comprehensive systematic review and meta-analysis of randomized controlled trials

**DOI:** 10.1371/journal.pone.0289172

**Published:** 2023-12-21

**Authors:** Jian-Ying Wang, Jiunn-Bey Pao, Chih-Hsin Lee, Jann-Yuan Wang, Ming-Chia Lee, Tzu-Tuan Wu

**Affiliations:** 1 Department of Pharmacy, New Taipei City Hospital, New Taipei City, Taiwan; 2 Section of Clinical Pharmacy, Department of Pharmacy, Taipei City Hospital, Taipei, Taiwan; 3 Department of Internal Medicine, Wan Fang Hospital, Taipei Medical University, Taipei, Taiwan; 4 Pulmonary Research Center, Wan Fang Hospital, Taipei Medical University, Taipei, Taiwan; 5 Division of Pulmonary Medicine, Department of Internal Medicine, Wan Fang Hospital, Taipei Medical University, Taipei, Taiwan; 6 Division of Pulmonary Medicine, Department of Internal Medicine, School of Medicine, College of Medicine, Taipei Medical University, Taipei, Taiwan; 7 Department of Internal Medicine, National Taiwan University Hospital, Taipei, Taiwan; 8 School of Pharmacy, College of Pharmacy, Taipei Medical University, Taipei, Taiwan; 9 Department of Nursing, Cardinal Tien College of Healthcare and Management, Taipei, Taiwan; 10 Department of Internal Medicine, New Taipei City Hospital, New Taipei City, Taiwan; AUSL della Romagna, ITALY

## Abstract

**Background:**

Olfactory dysfunction is a common manifestation in COVID-19 patients and can significantly impact their quality of life. Corticosteroids have been proposed as a potential treatment, but their efficacy remains controversial. This systematic review and meta-analysis aims to comprehensively analyze the efficacy of corticosteroid therapy for treating COVID-19-related olfactory dysfunction.

**Methods:**

A literature search was conducted in PubMed, Cochrane Library, and Embase databases up to March 1, 2023. Randomized controlled trials investigating the effects of corticosteroids on olfactory dysfunction in patients with COVID-19 were included. The primary outcome was the olfactory score at the end of follow-up, and the secondary outcomes were the duration and the rate of recovery from olfactory dysfunction.

**Results:**

Seven randomized controlled trials with 999 participants were included in the meta-analysis. Compared with the control group, corticosteroid treatment resulted in a statistically significant improvement in olfactory score with a standardized mean difference of 0.55 (95% CI: 0.15 to 0.95). Topical corticosteroids were found to be effective, but systemic corticosteroids were not. In addition, longer durations and higher dosages of corticosteroids treatment may also be associated with significant improvements in olfactory scores. No significant effect was observed on the duration or recovery rate of olfactory dysfunction.

**Conclusions:**

Our findings suggest that topical corticosteroid treatment is a viable option for improving COVID-19-related olfactory dysfunction, but further research is needed to investigate optimal treatment protocols and safety profiles.

## Introduction

The COVID-19 pandemic, caused by the SARS-CoV-2 virus, has had a significant impact on global health. As of March 2023, over 750 million confirmed cases and more than 6.8 million deaths have been reported worldwide [1]. One notable manifestation of COVID-19 is olfactory dysfunction, which affects approximately 47.85% of cases [2]. An early study reported that COVID-19-related olfactory dysfunction resolves spontaneously within two weeks in 95% of patients, with a mean recovery time of 9 days and 98% of patients recovering within 28 days [3]. However, recent studies suggest that approximately 25 to 33% of people inflicted with the SARS-CoV-2 virus continue to have some degree of measurable smell dysfunction for months after their infection [4–6]. With the large number of people affected by COVID-19, a substantial number of people worldwide will continue to experience olfactory dysfunction, leading to depression, anxiety, nutritional issues, and decreased quality of life.

Given that the mechanisms underlying COVID-19-related anosmia remain unclear, numerous theories have been proposed, including: nasal obstruction and rhinorrhea; mucosal edema in the olfactory cleft; damage to the olfactory epithelium (either directly or through immune-mediated mechanisms); injury to the olfactory bulb; and dysfunction of the central olfactory pathways [7–9]. Various interventions, including olfactory training and using corticosteroids, have been evaluated for the management of olfactory dysfunction in COVID-19 patients [10]. While olfactory training is a recommended intervention for COVID-19-related olfactory dysfunction, corticosteroids have been suggested as a potential treatment option due to their anti-inflammatory properties. Interestingly, parallels can be drawn to acute vestibular vertigo, a condition characterized by severe spinning sensation and nausea, caused by inflammation of the vestibular system [11]. Corticosteroids have been known to reduce vestibular inflammation, providing relief when administered within the first 1 to 2 days of symptom onset [11]. The anti-inflammatory properties of corticosteroids hint at their potential for managing COVID-19-related olfactory dysfunction, despite the ongoing debate about their efficacy [10, 12]. With nasal symptoms like nasal congestion and rhinorrhea possibly reflecting chronic inflammation in the nose and olfactory system, consideration of addressing nasal comorbidities such as allergic rhinitis or chronic rhinosinusitis may improve quality of life and aid in the recovery of smell function in long COVID patients [13, 14].

A recent meta-analysis [15], consisting of four randomized controlled trials (RCTs) and one cohort study revealed that topical corticosteroid treatment significantly improved olfactory scores compared with the control group. Subsequently, several studies have been published, and the results have shown inconsistent results when compared to the previous meta-analysis. Tragoonrungsea et al. indicated that no significant difference between corticosteroid nasal irrigation and saline or no irrigation in restoring the sense of smell in COVID-19-associated olfactory loss [16]. Hosseinpoor et al. concluded that there was no significant difference in olfactory scores, including smell test and visual analog scale (VAS) scores, between the corticosteroid nasal spray and control groups during the follow-up period [17]. However, based on the significant difference in olfactory score changes between the two groups, it appears that nasal corticosteroids may positively affect the recovery process of patients with COVID-19-related olfactory dysfunction who received treatment for more than 2 weeks. Additionally, Schepens et al. [18] showed that oral corticosteroid prednisolone did not improve olfactory function after COVID-19. Hence, we aim to conduct an updated meta-analysis with subgroup analysis to comprehensively examine the efficacy of corticosteroid therapy for COVID-19-related olfactory dysfunction. This was necessary due to the limited number of high-quality studies included in previous meta-analyses, inconsistent results from recent studies, and the need to identify a more suitable corticosteroid treatment regimen.

The PICOS framework was as follows:

- Population: Patients with COVID-19-related olfactory dysfunction.
- Intervention: Corticosteroid therapy.
- Comparison: Placebo or standard care.
- Outcome: Improvement in olfactory score
- Study design: Randomized controlled trials

## Methods

### Systematic literature review

This systematic review and meta-analysis was conducted following the Preferred Reporting Items for Systematic Reviews and Meta-Analyses (PRISMA) guidelines [19]. A literature search was performed in PubMed, Cochrane Library, and Embase databases from their inception to March 1, 2023, without language restrictions. The search terms used were: ("olfactory dysfunction" OR "anosmia") AND ("COVID-19" OR "SARS-CoV-2") AND ("corticosteroids" OR "steroids"). For detailed search strategy, please refer to S1 Table. Two independent reviewers screened all titles and abstracts for eligibility and identified relevant articles, and any discrepancies were resolved through discussion. The review protocol was prospectively registered in PROSPERO (registration number: CRD42023404491).

### Inclusion and exclusion criteria

This study only included RCTs that examined the effects of corticosteroids on olfactory dysfunction in COVID-19 patients. The exclusion criteria were studies with non-COVID-19-related olfactory dysfunction, studies that did not use corticosteroids as an intervention, and studies that did not report the outcomes of interest.

### Data extraction and quality assessment

Data were extracted independently by two reviewers using a standardized data extraction form. Information on study characteristics, participant characteristics, intervention type, and duration of treatment was extracted. The primary outcome of interest was the olfactory scores at the end of follow-up, which ranging from 0 as the worst to 10 as the best, and the secondary outcomes were the duration and recovery rate of olfactory dysfunction. We used risk ratio (RR) to calculate the effect sizes for the rate of recovery, mean difference (MD) to calculate the duration of recovery, and standardized mean difference (SMD) to calculate the olfactory scores due to the use of different measurement scales in different studies. The risk of bias for each RCT was assessed using the Cochrane risk of bias tool 2.0 (ROB 2.0) by two independent reviewers [20].

### Statistical analysis and data synthesis

We conducted a meta-analysis to evaluate the efficacy of corticosteroids in the treatment of olfactory dysfunction in COVID-19 patients. The pooled effect sizes were calculated using a random effects model, due to the anticipated heterogeneity in types, administration routes, and duration of use of corticosteroids. Heterogeneity was assessed using the Cochran’s Q test and the I^2^ statistic. Subgroup analyses were conducted to investigate the effect of treatment duration (≤2 weeks and >2 weeks), as suggested by the findings of Hosseinpoor’s study [17], as well as dose (high dose and normal dose) and route of administration (topical and systemic). We defined high dose corticosteroid nasal spray treatment as a dose that is greater than twice the defined daily dose (DDD) [21]. A sensitivity analysis was performed by omitting individual studies and investigating the effect of this omission on the overall results of the meta-analysis. Egger’s test was employed to evaluate the presence of potential publication bias in this analysis [22, 23]. All statistical analyses were performed using STATA 17.0 software.

## Results

### Eligible studies and patient characteristics

**Fig 1:**
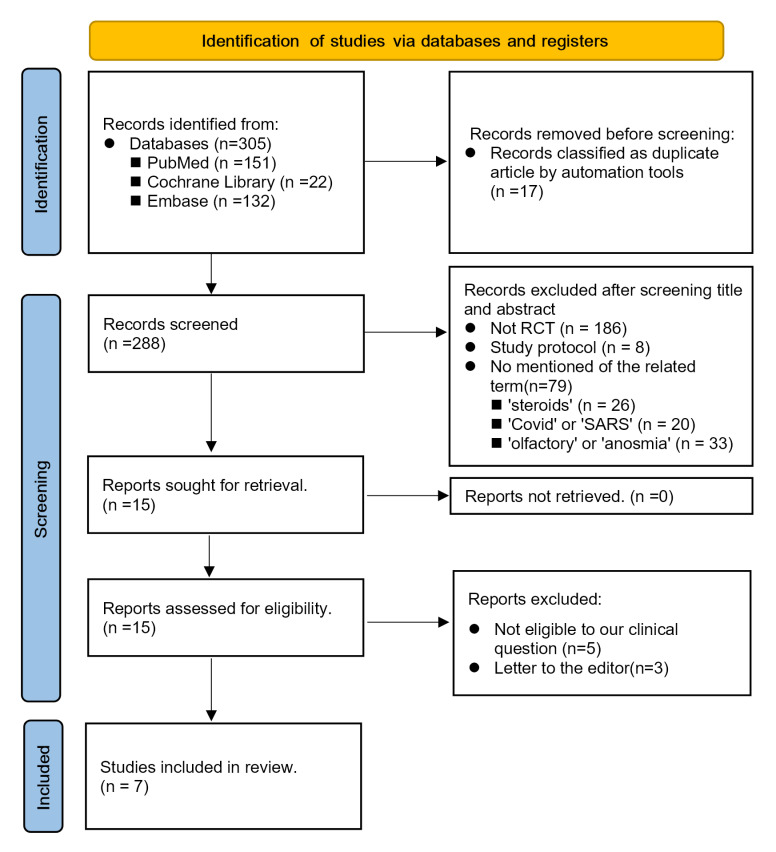
PRISMA flowchart depicting the selection process of studies for inclusion in the meta-analysis of the efficacy of corticosteroid therapy for COVID-19 related olfactory dysfunction.

The PRISMA flowchart of the study selection process was presented in Fig 1. Seven RCTs with a total of 999 participants, all of whom had olfactory dysfunction due to COVID-19 and were treated with corticosteroids [16–18, 24–27]. Of the seven studies included, six used topical corticosteroids [16–17, 24–27] and one study used systemic corticosteroids [18]. The duration of treatment varied across the studies, ranging from 10 days to 1 month. Participant enrollment in each study ranged from 70 to 276 individuals, with mean or median age from 29 to 49 years old. The proportion of male participants in each study ranged from 28.3% to 56.7%. Table 1 provides a summary of the characteristics of the included studies.

**Table 1:** Basic characteristics of the included studies.

Arthur, year	n	Age, mean (SD)	Male, n (%)	Participants	Intervention	control	Tx duration	follow-up
**Abdelalim, 2021 ** [25]	100	median:29.0 (IQR 21.75–38.0)	46 (46.0)	Patients aged 18 years or older who had a confirmed case of COVID-19 and had either recovered/discharged (with 2 negative PCR tests) or were currently experiencing sudden onset of anosmia or hyposmia with or without loss of taste.	MF nasal spray in an appropriate dose of 2 puff (100 μg) once daily in each nostril for 3 weeks with OT	OT	3 weeks	Week 1-3
**Hosseinpoor, 2022 ** [17]	70	33.6(11.3)	25 (35.7)	non‐hospitalized adult patients who had persistent anosmia or severe microsmia for more than 4 weeks due to COVID‐19 infection	one puff of 0.05% wt/vol MF intranasal spray on each side twice per day for 4 weeks	one puff of 0.65% wt/vol sodium chloride nasal spray	4 weeks	Week 2, 4
**Kasiri, 2021 ** [26]	77	35.4(9)	39 (50.6)	Adult patients diagnosed with COVID-19 through clinical findings and RT-PCR or lung CT scan results and had olfactory dysfunction symptoms for 2 weeks but were not hospitalized. Only those with severe anosmia or microsemia were chosen for the study based on UPSIT evaluation.	Two puffs of MF 0.05% nasal spray at an appropriate dose (100 μg) twice daily in each nostril for four weeks along with olfactory training	two puffs of topical saline spray in each nostril twice daily together with OT	4 weeks	Week 1-4
**Rashid, 2021 ** [27]	276	median:29 (IQR 23–37)	78 (28.3)	PCR-confirmed SARS-CoV-2 infection, age ≥ 18 years, and recent developed of anosmia.	3 drops for each nasal cavity 3-times daily of BSP drops (0.1 mg/mL) until recovery for a maximum of 1 month.	0.9% NaCl solution	up to 1 month	1 month
**Schepens, 2022 ** [18]	113	median:49 (IQR 41–57)	42 (37.2)	> 18 years old and if they had persistent (> 4 weeks) olfactory disorders within 12 weeks after a confirmed COVID-19 test.	oral 40 mg PRED once daily for 10 days + OT for 12 weeks	Placebo + OT for 12 weeks	10 days	Week 12
**Tragoonrungsea, 2023 ** [16]	213	34.3 (10.9)	90 (42.3)	COVID-19 patients >18 years old with new-onset of anosmia or hyposmia within 7 days confirmed the reduction in smell sensation scores on the olfactory self-assessment test	a mixture of 1 mg BD (1 mg/2 ml) with 500-ml of normal saline and rinse each nostril with 125-ml of solution twice daily for 2 weeks	saline IR or no treatment	2 weeks	Week 1-6
**Yildiz, 2022 ** [24]	150	38.5(10.5)	85 (56.7)	Patients aged 18-65, with other symptoms or with only acute odor loss, diagnosed with COVID-19 with RT-PCR	Saline IR (hypertonic solution/10 cc per nose, twice a day/1 month) plus nasal steroid spray 2*2puffs/each nose/TAC 0.055%).	No treatment or Saline IR	1 month	Days 15,30

Abbreviation: BD, Budesonide; BSP, Betamethasone Sodium Phosphate; CT, Computed Tomography; IQR, interquartile range; IR, Irrigation, MF, Mometasone furoate; OT, Olfactory training; PCR, Polymerase Chain Reaction; PRED, Prednisolone; RT-PCR, Real-time polymerase chain reaction; SD, Standard deviation; TAC, Triamcinolone acetonide; Tx, Treatment; UPSIT, The University of Pennsylvania Smell Identification Test.

### Effects of corticosteroids on olfactory dysfunction

**Fig 2:**
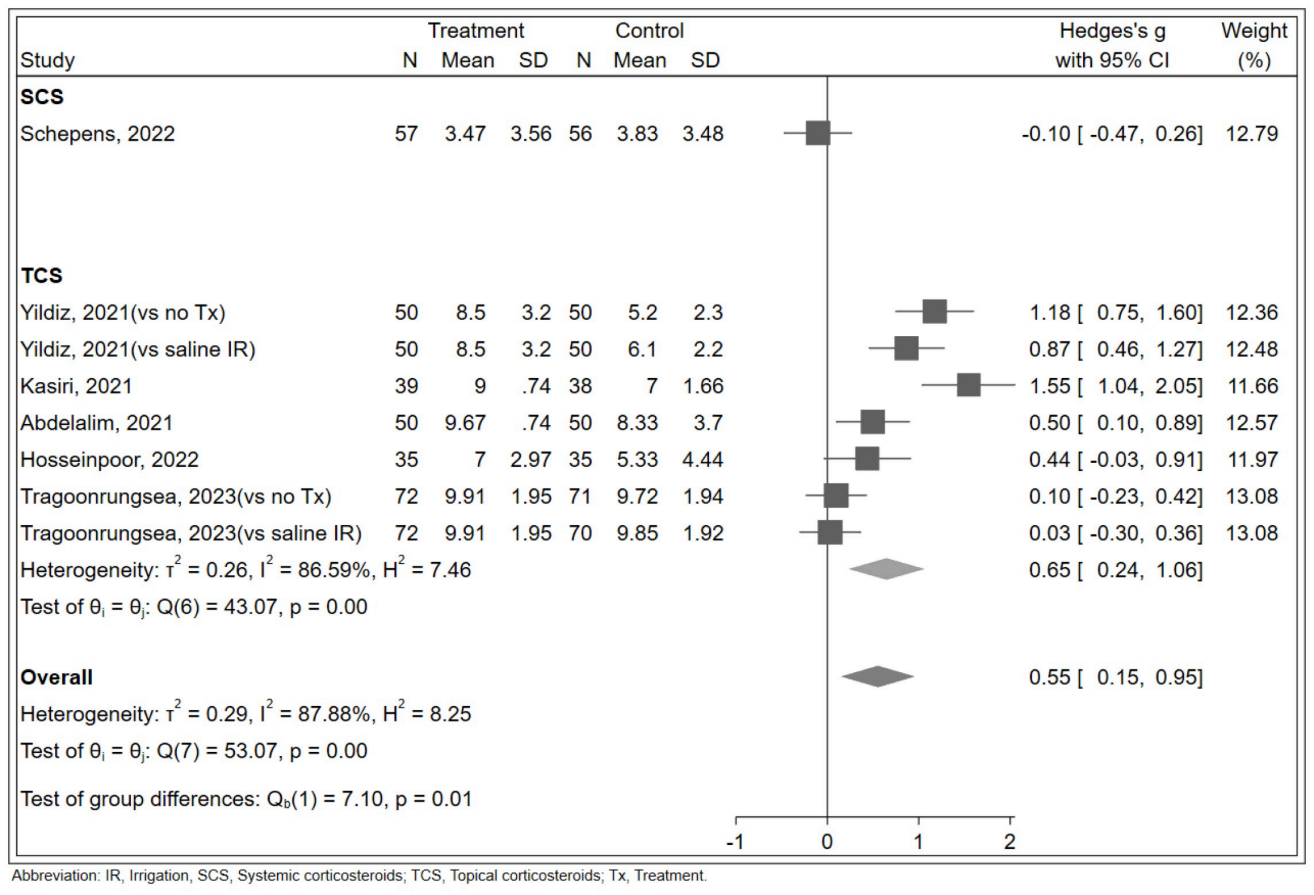
Forest plot showing the effects of corticosteroid therapy on olfactory scores in COVID-19 patients with olfactory dysfunction.

Regarding the primary outcome, our random-effects model demonstrated a statistically significant SMD of 0.55 (95% confidence interval [CI]: 0.15 to 0.95; Fig 2) in the olfactory score between the corticosteroid and control groups, indicating a positive impact of corticosteroid treatment on olfactory score improvement. However, the heterogeneity among the trials was high (I^2^ = 87.88%). The subgroup analysis showed that topical corticosteroids were more effective than systemic corticosteroids in improving olfactory scores, with an SMD of -0.10 and 0.65, respectively (p = 0.01). Additionally, the analysis revealed a significant difference between high dose and normal dose corticosteroid nasal spray treatment in improving olfactory scores (S1 Fig). Furthermore, treatment for more than two weeks was found to significantly improve olfactory scores compared to treatment for two weeks or less (S2 Fig). Taken together, these findings suggest that topical corticosteroids with longer treatment duration and higher dose may be more effective in improving olfactory dysfunction in COVID-19 patients. No effects on results of all outcomes were obtained for any study after performing a leave-one-out analysis.

**Fig 3:**
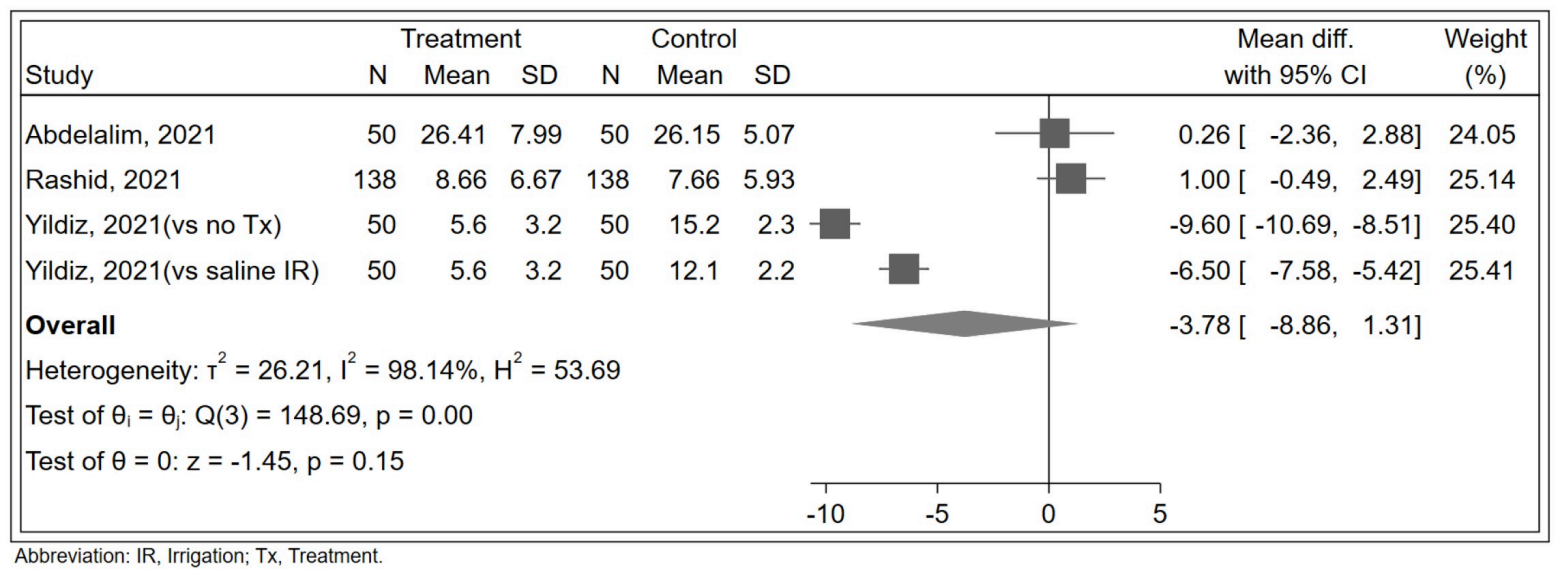
Forest plot showing the effects of corticosteroid therapy on the duration of recovery among COVID-19 patients with olfactory dysfunction.

**Fig 4:**
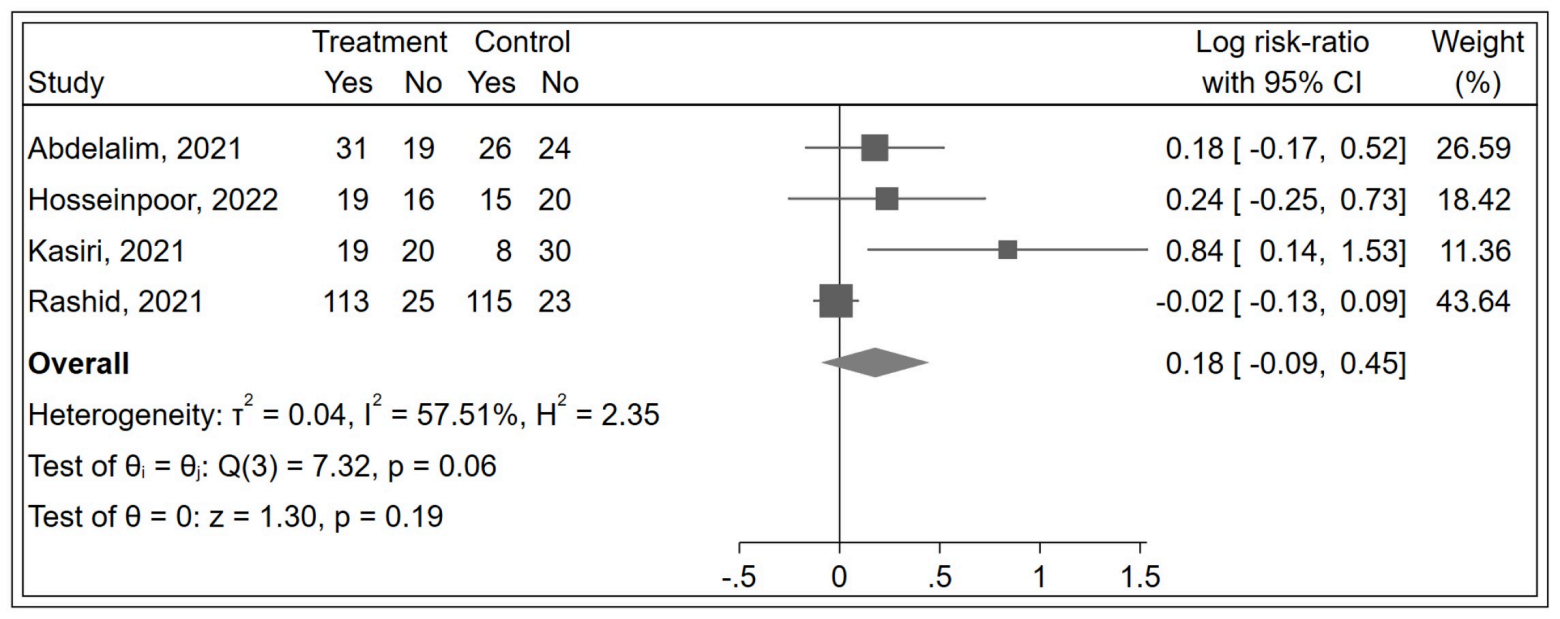
Forest plot showing the effects of corticosteroid therapy on the rate of recovery from olfactory dysfunction in COVID-19 patients.

For the secondary outcome, analysis from four studies indicated no significant impact of corticosteroids on either the duration (MD: -3.78 days; 95% CI: -8.86 to 1.31; Fig 3) or the recovery rate (Log RR: 0.18; 95% CI: -0.09 to 0.45; Fig 4) of olfactory dysfunction compared to controls. The limited number of studies reporting these outcomes may have reduced the power to detect significant effects.

Regarding safety profile, three out of the seven studies reported on the incidence of adverse effects associated with the use of topical corticosteroids. Hosseinpoor et al. [17] did not report any adverse reactions in either the intervention or control groups. In contrast, Schepens et al. [10] observed adverse effects in 9 out of 58 participants from the intervention group and in 5 out of 57 participants from the control group. However, none of these adverse events were severe enough to warrant discontinuation of the medication. Lastly, Tragoonrungsea et al. [16] identified that corticosteroids might reduce the risk of nasal dryness, based on a comparison of adverse events such as pharyngeal burning, nasal burning, pharyngeal dryness, and nasal dryness. There were no significant differences in the other three events between the groups. Despite the limited number of studies, these findings collectively suggest that topical corticosteroids could potentially have a favorable safety profile in the management of olfactory dysfunction in COVID-19 patients.

### Quality assessment

**Fig 5:**
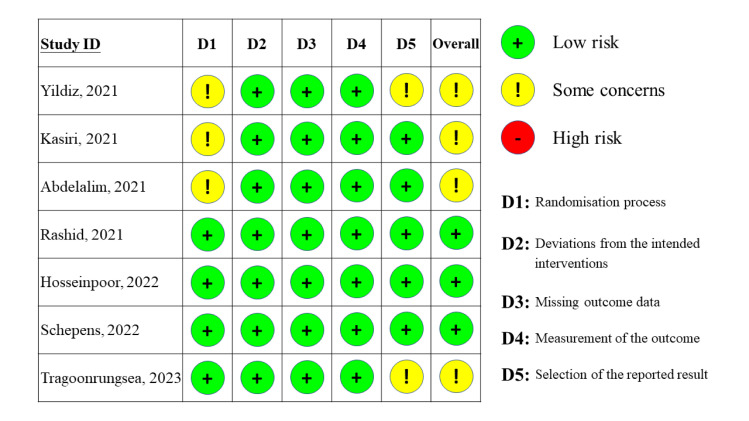
Risk of bias graph depicting the assessment of risk of bias in the included studies using the Cochrane risk of bias tool 2.0.

A risk of bias graph was presented in Fig 5, showing that three of the seven studies had a low risk of overall bias, while the remaining four were rated as having some concerns. Among the studies with some concerns, three were flagged for issues related to the domain of random sequence generation or allocation concealment, while two studies had concerns related to the risk of reporting selected outcomes.

For publication bias, an Egger test was conducted to assess the presence of small-study effects and potential publication bias. The results indicated that there was no significant evidence of small-study effects for the duration of olfactory dysfunction (p = 0.16). However, there was significant evidence of small-study effects for the olfactory score and the rate of recovery from olfactory dysfunction (p = 0.001 and p = 0.01, respectively).

## Discussion

Our study provides important evidence for clinical professionals in making decisions regarding the use of corticosteroid treatment for COVID-19-related olfactory dysfunction. COVID-19 has been affecting people worldwide for a significant amount of time, leading to a substantial impact on individuals’ quality of life. Currently, there are limited treatment options available for COVID-19-related olfactory dysfunction [28]. The results of our analysis suggest that corticosteroid treatment is effective in improving olfactory dysfunction in COVID-19 patients. Specifically, we found a statistically significant improvement in olfactory scores with corticosteroid treatment, with a SMD of 0.55 (95% CI: 0.15 to 0.95). Although heterogeneity among the studies was high, a sensitivity analysis indicated that our findings are robust and not overly influenced by any single study. In addition, we found that the included studies had a low risk of bias, which further strengthens the reliability of our findings.

The subgroup analysis indicated that topical corticosteroids, but not systemic corticosteroids are effective in improving olfactory scores. However, this result contrasted with the findings of a small randomized controlled trial conducted by Cheema et al. [29]. This trial was not included in our analysis because it compared the efficacy of systemic and topical corticosteroids in improving olfactory dysfunction in COVID-19 patients, rather than comparing corticosteroid treatment with a control group. The study reported that olfaction was regained earlier in patients treated with systemic steroids compared with topical steroids (p < 0.001). It is crucial to emphasize that our analysis did not primarily focus on comparing the effectiveness of oral and nasal administration of corticosteroids in improving olfaction. Due to the limited number of studies examining the efficacy of systemic corticosteroids in treating olfactory dysfunction, it is currently premature to draw any conclusions about their effectiveness. However, it is worth noting that if topical corticosteroids are effective, they may be a preferred treatment option due to their lower likelihood of producing systemic side effects. Moreover, our subgroup analysis revealed a statistically significant difference in olfactory scores between high dose and normal dose corticosteroid nasal spray treatment for COVID-19-related olfactory dysfunction. This suggests that higher doses of corticosteroid treatment may be more effective in improving olfactory dysfunction in COVID-19 patients. In addition, longer duration of treatment was associated with a significant improvement in olfactory scores compared to shorter treatment duration. Therefore, our analysis suggests that using topical corticosteroids at a higher dose, equal to or greater than 2 times DDD, for a duration longer than two weeks, may lead to greater improvement for COVID-19-related olfactory dysfunction. Additionally, as topical corticosteroids have a lower risk of producing systemic side effects, they may be a safer option compared to systemic corticosteroids. Due to safety concerns, several publications have advised against prescribing systemic corticosteroids, particularly in active cases [30–32]. Hence, it is advisable to conduct a thorough evaluation of the patient’s underlying condition, existing comorbidities, imaging findings, and perform a comprehensive risk-benefit assessment before administering oral or topical corticosteroid therapy. Our findings provide evidence to support the use of topical corticosteroids as a therapeutic option for patients with persistent COVID-19-related olfactory dysfunction, especially for those distressed by lingering smell loss. Clinicians can consider prescribing a trial of high-dose nasal corticosteroids for 2 to 4 weeks to determine if a patient experiences subjective or objective improvement in smell function.

A recent meta-analysis conducted by Kim et al. included one cohort study and four randomized controlled trials up to September 2021 [15]. Their results showed that topical steroids improved acute-onset olfactory dysfunction caused by COVID-19, but there was no significant difference in the rate of full olfactory recovery between treated and control patients, which is consistent with our findings. However, our study provides additional insights as we searched up until March 2023, including more recent literature, and performed subgroup analysis on the effects of treatment duration and high-dose versus normal-dose corticosteroid nasal spray treatment, as well as analyzing the recovery time. Furthermore, it is important to note that our study focused specifically on the effects demonstrated by randomized controlled trials.

While our study provides valuable insights into the use of corticosteroids for COVID-19-related olfactory dysfunction, it is important to acknowledge its limitations. Firstly, the number of RCTs included in our meta-analysis was relatively small, which may limit the generalizability of our findings. Secondly, there was variation in the dosage and duration of corticosteroid treatment among the included studies, which contributes to high heterogeneity and makes it difficult to determine the optimal treatment protocol for this condition. Although sensitivity analysis did not affect our overall results, such heterogeneity introduces uncertainty to our findings and may have biased our overall effect size estimate. Furthermore, differences in patient populations across the included studies, such as age, severity of olfactory dysfunction, time-to-treatment, and severity of COVID-19, might have an influence on the outcomes. Despite the possibility of conducting stratified analyses, the high data heterogeneity and limited number of studies could have led to insignificant or unreliable results. Thus, these factors warrant careful consideration in future studies aiming to deepen our understanding of corticosteroid effectiveness for COVID-19-related olfactory dysfunction. Additionally, we detected publication bias in two of our outcomes, suggesting that selective reporting of studies with positive results could potentially lead to overestimation of the treatment effect. Moreover, some studies included in our analysis did not report results concerning adverse reactions, which limits our understanding of the safety profile associated with corticosteroid treatment in COVID-19 patients, particularly those with underlying comorbidities or who are taking other medications. Considering the potential of corticosteroids to suppress the immune system, especially in high doses or long-term use, further research on their safety profile in this population is crucial. Furthermore, the limited number of studies reporting the time for olfactory dysfunction recovery or the rate of recovery from olfactory dysfunction could have limited our ability to detect a significant effect of corticosteroid treatment on these outcomes. This underscores the need for future research to overcome these limitations, including larger RCTs to validate our findings. Additionally, more investigation into optimal treatment protocols, as well as long-term efficacy and safety of corticosteroid treatment, is crucial. Such rigorous studies will provide further insights into the potential benefits of corticosteroids for olfactory dysfunction in COVID-19 patients.

## Conclusion

In summary, topical corticosteroids may be beneficial for improving olfactory dysfunction from COVID-19, especially when used at higher doses and for longer durations. However, larger, high-quality studies are needed to confirm these findings, determine optimal treatment regimens, including precise dosage and duration, and evaluate long-term efficacy and safety.

## Supporting information

S1 Table Search strategy and results. (DOCX)

S1 Fig Forest plots of the effects of high dose and normal dose corticosteroid nasal spray treatment on changes in olfactory scores for COVID-19 patients with olfactory dysfunction. (TIF)

S2 Fig Forest plots of the effects of different treatment durations on changes in olfactory scores for COVID-19 patients with olfactory dysfunction. Studies were categorized into those with treatment durations of ≤ 2 weeks and those with treatment durations of > 2 weeks.
(TIF)

S1 File PRISMA checklist. (DOCX)
