# Supplementary material for: Corticosteroids for COVID-19-induced olfactory dysfunction: A comprehensive systematic review and meta-analysis of randomized controlled trials
Source: PLoS One. 2023 Dec 21;18(12):e0289172. doi: 10.1371/journal.pone.0289172 (PMC10734960; doi:10.1371/journal.pone.0289172)
Supplement: S1 Table — (DOCX) [file pone.0289172.s001.docx]

**S1 Table.**

| Database | Search strategy | Results |
| --- | --- | --- |
| PUBMED | #1. olfactory dysfunction[All Fields] | 8,583 |
|  | #2. olfactory[All Fields] | 68,470 |
|  | #3. anosmia[All Fields] | 4,449 |
|  | #4. COVID-19[All Fields] | 348,564 |
|  | #5. SARS-CoV-2[All Fields] | 196,231 |
|  | #6. corticosteroid[All Fields] | 379,041 |
|  | #7. steroid[All Fields] | 1,138,687 |
|  | #8. (#1 OR #2 OR #3) AND (#4 OR #5) AND (#6 OR #7) | 151 |
| Cochrane Library | #1. olfactory dysfunction, in Trials | 238 |
|  | #2. olfactory, in Trials | 1334 |
|  | #3. anosmia, in Trials | 403 |
|  | #4. COVID-19, in Trials | 14,416 |
|  | #5. SARS-CoV-2, in Trials | 452 |
|  | #6. corticosteroid, in Trials | 14702 |
|  | #7. steroid, in Trials | 17192 |
|  | #8. (#1 OR #2 OR #3) AND (#4 OR #5) AND (#6 OR #7), in Trials | 22 |
| EMBASE | #1. olfactory AND dysfunction | 5,667 |
|  | #2. olfactory | 76,669 |
|  | #3. anosmia | 11,498 |
|  | #4. COVID-19 | 315,486 |
|  | #5. SARS-CoV-2 | 130,855 |
|  | #6. corticosteroid | 356,065 |
|  | #7. steroid | 407,069 |
|  | #8. (#1 OR #2 OR #3) AND (#4 OR #5) AND (#6 OR #7) | 640 |
|  | #9. #8 AND 'clinical article'/de AND 'article'/it | 132 |
