# Supplementary figures and images for: Corticosteroids for COVID-19-induced olfactory dysfunction: A comprehensive systematic review and meta-analysis of randomized controlled trials
Source: PLoS One. 2023 Dec 21;18(12):e0289172. doi: 10.1371/journal.pone.0289172 (PMC10734960; doi:10.1371/journal.pone.0289172)

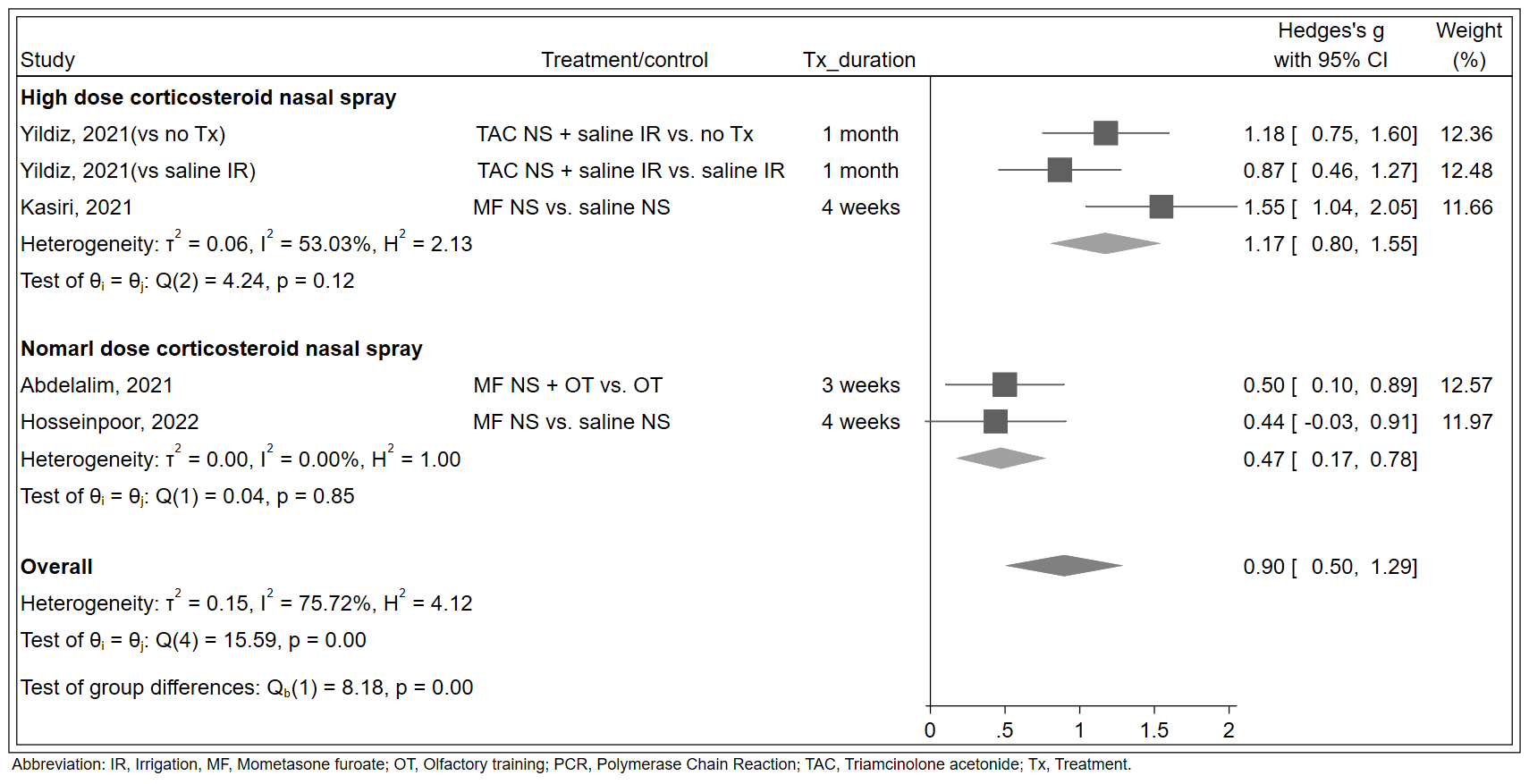

Supplement: S1 Fig — (TIF) [file pone.0289172.s002.tif]

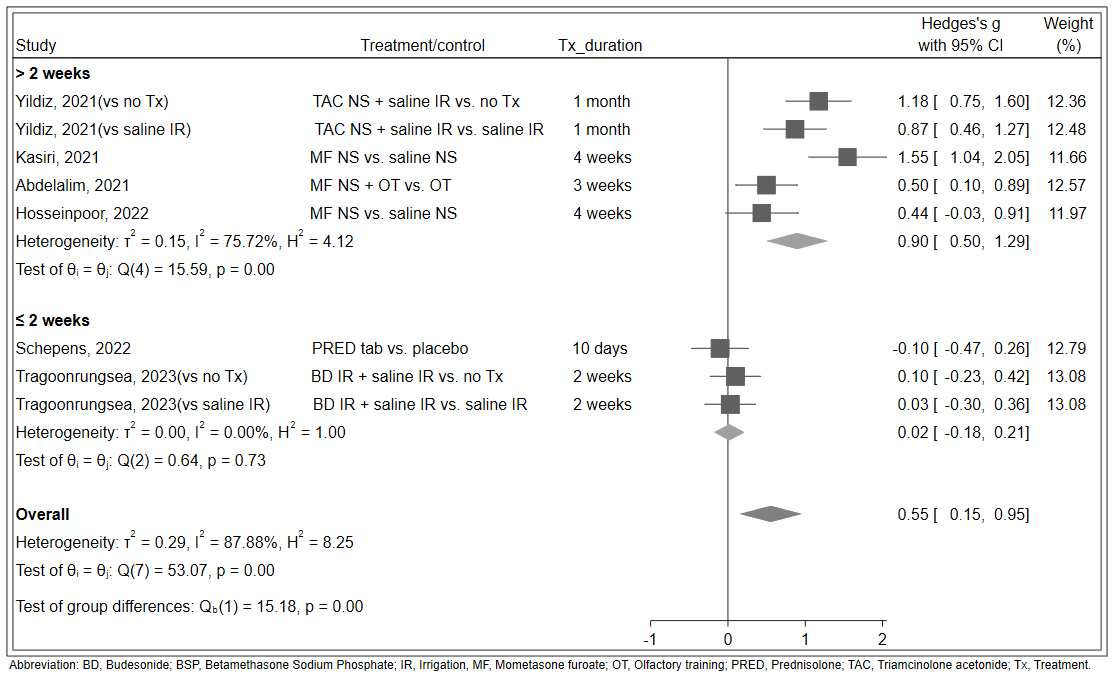

Supplement: S2 Fig — Studies were categorized into those with treatment durations of ≤ 2 weeks and those with treatment durations of > 2 weeks. (TIF) [file pone.0289172.s003.tif]
